# National Sanitary Legislation

**Published:** 1898-12

**Authors:** 


### EDITORIAL DEPARTMENT.

F. E. DANIEL, M. D., Editor.
S. E. HUDSON, M. D., Managing Editor.
A. J. SMITH. M. D., Galveston, Associate Editor.

Published Monthly at Austin, Texas, by Drs. Daniel and Hudson. Subscription price $1.00 a year in advance.

Eastern Representative: John Guy Monihan, St. Paul Building, 220 Broadway New York City.

Official organ of the West Texas Medical Association, the Houston District Medical Association, the Austin District Medical Society, the Brazos Valley Medical Association, the Galveston County Medical Society, and several others.

### National Sanitary Legislation.

Congress met at 12 o'clock, December 5th (inst.). It is likely that some action will be taken on the several bills looking to a change in the Federal quarantine laws. Our readers are urged to write to Senators and Representatives to oppose the Caffery bill. What is known as the Spooner bill has been endorsed by the American Medical Association and by the American Public Health Association. It provides for a Commissioner, or other head of the department, and an advisory board composed of one representative from each State—forty-five in all—the whole to be *under the wing of the Secretary of the Treasury!* That clause ruins it.

The momentous interests involved in a consideration of the question of public health—for the commerce of the country is inseparably connected with it—would seem to justify, if not to demand, a separate Department of Public Health, and the Commissioner or Secretary should have equal dignity and rank with the heads of other departments—a seat in the President's Cabinet.

The most zealous advocate of State's rights must admit that it is the duty of the general government to protect the States from foreign invasion as well by infection as by armed force; and to this end there should be some one at the head of our sanitary interests authorized and empowered to bring about international agreement and co-operation. Inspections by reliable and competent men at each port of departure where infection exists, is the first and most important step. No infected person or thing should be allowed to leave any port for the United States; no person, whether he has been directly exposed or not, should be permitted to sail, within the incubative period of yellow fever, and all baggage should be sterilized.

The system of quarantine should be revolutionized. Instead of all ports shutting their doors against an infected place, the infected place should be cut off from the outside world, and the infection eradicated. However, this is a mere detail. The right kind of a man at the head of a Department of Public Health would soon secure the proper legislation, and institute the proper measures for dealing with this question.

IN THIS connection, we notice a vigorous protest in the *New Orleans Picayune* against national control of quarantine. In an address by Mr. F. C. Zacharie, a noted lawyer of that city, before the Credit Men's Association, the relation of commerce to quarantine is fully discussed, and it is shown how vital is that relation. Mr. Zacharie goes back in history as far as 1745, and reviews the struggle between the East and the South to secure the trade of the Mississippi valley. He shows what efforts have been made in former years, and what sums have been expended to divert it to the Atlantic ports, and he assumes—by what right is not shown—that the head of a National Quarantine Department could not withstand the pressure that would be brought to bear to discriminate against the Southern ports. He says: "When we contemplate the powerful aggregations of capital represented in the Vanderbilt system, the Gould system, the Baltimore and Ohio, the Pennsylvania Central, the Morgan syndicate, the Northern, Central and Union Pacific Railroad syndicates, the combined wealth of the great steamship lines from the Eastern ports, all amounting to billions of dollars, and the powerful army of their organized lobbyists always alert and vigilant at the national government to influence and pervert Federal action to their commercial advantage, and to the disadvantage of their commercial rivals, we may well pause and consider the effect and influence of this powerful combination on one man who will hold in the hollow of his hand the whole quarantine power of the land, not to speak of its influence on his hundreds of employees who will owe no allegiance to local interests, but will only execute the will of their official head."

In this Mr. Zacharie goes most too far, we think. While there is much force and truth in what he says, still we have faith enough in the medical profession to believe that a man, perfectly competent to fill the great and responsible position of Commissioner or Secretary of Public Health with credit, success, honor and dignity, can be found, who is incorrupt and incorruptible. At least some change should be tried. The inefficiency of the present method—a Bureau of Health as a sub-department of the Treasury Department—has been abundantly demonstrated, and the situation demands a change. We are still in favor of State control of State quarantine, but a National Health and Sanitary Department is an essential, as a protection, in the first place, against the *importation of infection.* Were this protection afforded, State quarantine on the coast would not be necessary; and, so long as it *is* necessary, and our ports are shut against "all places south of 25 degrees north latitude" from May till November, we do not clearly see what difference it makes whether those ports are blockaded by National or State quarantine; they are blockaded every year by State quarantine.

Let us have a modification of the Spooner bill; a bill creating a Department of Public Health, with a Secretary a Cabinet officer, and with the relation of State health authorities thereto properly adjusted, each State left free to exercise its quarantine rights, which, together with all other police rights and powers, are, by numerous decisions, shown to be reserved to the States under the Constitution—never having been delegated to the general government, and which, with any decent regard for the Constitution, cannot be usurped by the National government.

